# A knowledgebase system to enhance scientific discovery: Telemakus

**DOI:** 10.1186/1742-5581-1-2

**Published:** 2004-09-21

**Authors:** Sherrilynne S Fuller, Debra Revere, Paul F Bugni, George M Martin

**Affiliations:** 1 Telemakus Research Program, Division of Biomedical & Health Informatics, University of Washington, Seattle, WA, USA; 2 Department of Pathology, School of Medicine, University of Washington, Seattle, WA, USA

## Abstract

**Background:**

With the rapid expansion of scientific research, the ability to effectively find or integrate new domain knowledge in the sciences is proving increasingly difficult. Efforts to improve and speed up scientific discovery are being explored on a number of fronts. However, much of this work is based on traditional search and retrieval approaches and the bibliographic citation presentation format remains unchanged.

**Methods:**

Case study.

**Results:**

The Telemakus KnowledgeBase System provides flexible new tools for creating knowledgebases to facilitate retrieval and review of scientific research reports. In formalizing the representation of the research methods and results of scientific reports, Telemakus offers a potential strategy to enhance the scientific discovery process. While other research has demonstrated that aggregating and analyzing research findings across domains augments knowledge discovery, the Telemakus system is unique in combining document surrogates with interactive concept maps of linked relationships across groups of research reports.

**Conclusion:**

Based on how scientists conduct research and read the literature, the Telemakus KnowledgeBase System brings together three innovations in analyzing, displaying and summarizing research reports across a domain: (1) research report schema, a document surrogate of extracted research methods and findings presented in a consistent and structured schema format which mimics the research process itself and provides a high-level surrogate to facilitate searching and rapid review of retrieved documents; (2) research findings, used to index the documents, allowing searchers to request, for example, research studies which have studied the relationship between neoplasms and vitamin E; and (3) visual exploration interface of linked relationships for interactive querying of research findings across the knowledgebase and graphical displays of what is known as well as, through gaps in the map, what is yet to be tested. The rationale and system architecture are described and plans for the future are discussed.

## Background

An unfortunate consequence of specialization in the sciences is poor communication across research domains – which can hamper the knowledge discovery process. Research findings in one area may be pertinent to another, researchers may be unaware of relevant work by others that could be integrated into their work and important findings just outside a researcher's focus can be overlooked. Compounding this problem is the difficulty of keeping current with new research findings that continue to grow at an exponential rate.

Reliance on keywords and/or subject indexing to find relevant literature limits the researcher's ability to precisely search for and locate specific research findings. For example, a typical database query to locate all research articles reporting a statistically significant relationship between caloric restriction and cancer would retrieve articles reporting both concepts as represented by the indexing and keyword search – but not necessarily linked together as a research finding, with information regarding reported statistical significance of the finding, nor, perhaps most importantly, lacking representation of the linkages among the retrieved document sets.

This lack of "interactivity" among retrieved citations is a critical limitation of traditional search and retrieval systems. As stated by Swanson (1986) in his examination of "mutually isolated literatures:"

"Knowledge can be public, yet undiscovered, if independently created fragments are logically related but never retrieved, brought together, and interpreted [..] This essential incompleteness of search and retrieval therefore makes possible, and plausible, the existence of undiscovered public knowledge [1]."

In addition to this limitation of search and retrieval, there are questions about representing a set of documents: What format or display of the retrieval set most enhances users' ability to identify which documents need to be examined in more detail? How can users navigate across document sets (i.e., to explore linkages) to enhance the discovery process? The bibliographic citation format is used by virtually all bibliographic databases today to report the results of database searches. However, it does not provide a way for the user to quickly review retrieved results for research methods and findings or to quickly view the relationships among the documents in the document set. Abstracts, even structured abstracts, simply do not provide a format conducive to rapid review of retrieved citations. In fact, the bibliographic citation format itself has changed little for the past two hundred years – even though it does not present an accurate representation of either the research methods or the research findings in a document [2].

In spite of great improvements in document retrieval over the past twenty years, most information systems developed to promote scientific discovery (e.g., [3-6]), are based on traditional search and retrieval approaches and the tools for locating and inter-relating research methods and findings are imprecise. This is the impoverished state Nobel laureate economist Herbert Simon described in his oft-cited remark on information as a commodity: "What information consumes is rather obvious: it consumes the attention of its recipients. Hence, a wealth of information creates a poverty of attention and a need to allocate that attention efficiently among the overabundance of information sources that might consume it" [7]. For researchers, such a poverty of attention can translate into delays in the progress of scientific discovery.

A comprehensive approach to these challenges is the goal of the Telemakus research program. Telemakus was named for the son of Odysseus who went searching for his father, the legendary Greek hero of Troy. Similarly, the Telemakus research program is developing approaches and tools for searching, knowledge discovery and mapping domain knowledge. The overall vision is to enhance the knowledge discovery process through retrieval, visual and interactive interfaces and tools. In close collaboration with researchers in the biology of aging, a working knowledgebase system has been designed to present aggregated citation information and research methods and findings for display in a conceptual schema.

The Telemakus KnowledgeBase System provides the user with both a macro- and micro-view of domain knowledge. The macro-view facilitates identification of patterns – both expected and unexpected occurrences of relationships among research concepts – and permits visualization and dynamic navigation of scientific domains. The resulting maps are analogous to citation mapping work done by Small [8] but, instead of citations, rely on reported research findings. The micro-view presents consistent and detailed document attributes including research methods and findings for each document in the database.

This article describes the theories underlying the Telemakus KnowledgeBase System, provides an overview of its implementation, reports initial user feedback and explores future directions. Telemakus system builds on prior research in the areas of: (1) schema theory, (2) concept representation and (3) information visualization.

### Schema Theory

Schemas are generalized mental models that provide a guide for structuring the process of production and comprehension of texts: "...at the simplest level, a schema is a description of a complex object, situation, process or structure. It is a collection of knowledge related to the concept [9]." According to schema theory, we understand the world in terms of prototypical patterns: people capture global coherence or structure their knowledge of the world based on scripts, schemas and narratives in which are embedded a vast array of relationships, concepts and vocabulary words. Individuals not only think and store knowledge in terms of scripts, frames and schemas but they also produce texts this way. Schema theory originated in linguistics and cognitive psychology as a model for holistically representing texts; it is primarily concerned with the "glue" which holds texts together, i.e., grammatical markers that allow texts to be cohesive as well as coherent [10].

"The crucial fact is that the cognitive constraints on information processing which require the formation of semantic macro-structures and which organize acts and speech acts in global units, at the same time have social implications: they determine how individuals wish, decide, intend and plan, execute and control, "see" and understand their own and others' speaking and acting in the social context. Without them the individual would be lost among a myriad of detailed incoherent pieces of visual, actional and prepositional information [10]."

Research on the application of schema theory to scientific research includes the schematic representation of psychological reports [11], clinical trials [12,13] and, more recently, Dillon's work on the superstructure and predictability of text [14]. An elaboration and refinement of schemas are frames [15]. Like schemas, a frame is a data-structure for representing a stereotyped situation, a remembered framework to be adapted to fit reality by changing details as necessary. When one encounters a new situation (or makes a substantial change in one's view of a problem) one selects from a memory structure called a frame. "Attached to each frame are a number of kinds of information. Some of this information is about how to use the frame. Some of it is about what one can expect to happen next. Some is about what to do if expectations are not confirmed [15]." The example commonly used is entering a restaurant: If the tables have checkered tablecloths and paper napkin dispensers, we assume that the prices on the menu will be lower than at a restaurant with white linen tablecloths and napkins rolled around polished silverware.

Understanding a written text is a process of fitting it into a larger schema known to reader as part of their previous knowledge about the world. It is reasonable to expect that presenting written texts in familiar formats can enhance and potentially speed up an individual's ability to review and analyze large document sets rapidly.

Fuller [13] investigated the application of schema theory in the symbolic representation of full-text research reports to improve representation of research findings. She concluded that schema analysis offered promise for representing the document structure above the level of individual words and sentences and that such schemas, "...offer a means of writing precise computer programs in terms of the specific schema elements, based on the portions of the document where they are most likely to occur. [..] Schema theory appears to offer a paradigm or framework for indexing which provides a means of capturing both the intra-document (i.e., research design, methods and outcomes) and inter-document relationships. [..] It seems likely that the schema will prove effective as a means of improving the retrieval of documents both in terms of precision and relevance [13]."

The predictability provided by schemas also applies to a document's metastructure. For example, Dillon [16] has proposed a model of navigation in electronic environments which assumes that experienced users of information form schematic representations of a document that in part represent its layout and structure. The Telemakus system utilizes the inherent and predictable research report layout and structure to create schematic representations or surrogates of research studies with extracted representations of research environment, methods and outcomes [17,18].

A second core component of the Telemakus system is based on concept representation.

### Concept Representation

Concept representation is an important component in accurately representing facts from the document. Characterizing the location of concepts in a scientific document can greatly facilitate accurate document representation.

Indexing a document – using a vocabulary or thesaurus of terms to represent the document – is a standard method employed to improve retrieval of relevant documents. Yet traditional approaches to indexing fall short of true document representation: reducing the words found in the abstract, title or full-text of the document may be suggestive of the content but are not truly representative of the methods and research findings. The indexing literature is replete with studies documenting interindexer inconsistency, even among experienced professionals using familiar well-documented systems [19]. Studies indicate that human indexers usually select the most frequently occurring words in a document, yet they will disagree on the terms used and the same indexer will use different terms to index the same document at different times [20]. Many current automated retrieval programs also rely on word frequency, thus equating frequency with importance for retrieval purposes, which may be a faulty assumption [21].

"The information retrieval (IR) problem can be described as a quest to find the set of relevant information objects (i.e., documents D) corresponding to a given information need, represented by a query Q. The assumption is that the query Q is a good description of the information need N. An often used premise in IR is the following: if a given document D is about the request Q, then there is a high likelihood that D will be relevant with respect to the associated information need. Thus the information retrieval problem is reduced to deciding the aboutness relation between documents and queries [22]."

Another problem with current indexing practice lies in the way the unique structure of the information elements in the document is obscured. Scientific research reports have a highly predictable structure, with an introduction, methods, results and conclusion. Concepts mentioned in the introduction or conclusion section of a scientific article may not be the primary focus of the research described within the document. For example, the Introduction may include discussion of research among several animal models whereas the target of the research study itself is a specific breed of mouse. However, current indexing processes (whether human or automated) rarely discriminate between locations of concepts in the document for indexing purposes. In addition, index terms do not represent the connections between the various elements in the document; thus, a significant amount of critical information for the scientist is lost.

For example, it is not possible to unambiguously retrieve citations from PubMed^®^ or any other bibliographic database today that will answer the question: "Has anyone ever published data that supports a connection between cancer and caloric restriction? If so, what was the intervention, what type of experiments were done and what were the findings?" A successful response to a query of this type is extremely difficult or impossible in traditional information retrieval systems because: " [..] conventional IR systems that employ isolated term assignments seem inadequate for queries which are specific and empirical in nature. If, on the other hand, retrieval systems provide a link to represent the relationships between the variables of interest as reported in the documents, queries [..] would be better answered. That is, precision might be enhanced for specific and empirical queries when the relationships between the index terms were specified in retrieval systems [23]."

In other words, the researcher asking the questions above can retrieve a set of citations that contain both topics but still must go through the full-text of each document to determine if the research specifically answers the question.

Several research studies have explored the utility of relationships captured from data tables and figures in scientific research studies. Fuller, et. al. [24] described the application of the relationship analysis process for quality filtering of the scientific literature and found it compared favorably with other measures of quality, including the Science Citation Index Impact Factor. Weiner, et. al. [25,26] applied relationship analysis and a mapping method to represent research findings from a database of cancer studies and found they could identify directions for new research studies. And Yamaguchi, et. al. [27] studied the relative importance of quantitative ideas as expressed in sentences in the text of the Results sections of research reports and in the data tables and concluded that the text ideas were more difficult to find and extract and were found to be less important when compared with ideas derived from the data tables.

The importance of data tables for expert decision-making was underscored by Malogolowkin, et. al. who found that cancer researchers rely on ideas presented in numerical displays in published research studies for much of their design of new research protocols [28]. Malogolowkin, et. al. concluded that innovative aspects of the design can be traced and better understood by mapping the numeric relationships [28].

Oh [23,29,30] investigated the utility of empirical variables and their associated statistical relationships in document representation and retrieval and designed an empirical fact retrieval system (EMFRS). Results of the evaluation indicated that the EMFRS generally outperformed the traditional retrieval system in terms of precision, search effort and measures of user satisfaction.

Identifying semantic relationships in text involves looking for certain linguistic patterns in the text that indicate the presence of a particular relationship (or research finding) using pattern-matching to identify the segments of the text or the parts of the sentence that match with each pattern: "If semantic relationships can be identified accurately in the text, retrieval results can be improved by eliminating documents containing the required concepts but not the desired relationships between the concepts [31]."

The third component of the Telemakus system is based on visual mapping of reported research findings.

### Mapping Inter- and Intra-document Relationships

As previously mentioned, indexing strategies rely on "isolated term assignments." This approach leads to the loss of two important sources of information: (1) intra-document information, i.e., the research relationships studied and tested and (2) cross-document information, which captures and links research relationships across groups of documents or domains. This loss is the result of breaking apart the context of clearly linked in research findings in the data tables and figures, concepts typically linked together (the x-y axes of the tables and graphs).

Once the research relationships have been extracted, concept mapping, a means of spatially representing knowledge in a visual format, provides a potential solution to the challenge of maintaining the inter-relationships between documents and reported research findings. Spatial representations can assist in understanding conceptual relationships across a domain. They can also assist in identifying previously overlooked potential research connections.

Numerous approaches to visualizing an information retrieval space have been explored (e.g., [8,32,33]), all seeking to capitalize on the natural strength people have for rapid visual pattern recognition. Most mapping work to date has focused on similarity between journal articles using citation analysis [8], co-occurrence or co-classification using keywords, topics, or classification schemes [34-36], or journal citation patterns [37]. Latent semantic analysis (LSA) has been used to map co-occurrence of words (or authors) in titles, abstracts, or full-text sources [33] and domain maps have been used to visualize author co-citation analysis [32].

While a review of information visualization strategies is outside the scope of this paper, there is a growing body of work related to mapping metaphors and visualizing large document sets and database search results to provide the user with the ability to visualize relationships among documents and their contents [38,39]. In addition, several tools have been developed that graphically present inter-document relationships, most commonly using some form of link-node diagram [40,41].

Concept mapping represents knowledge graphically through networks of ideas. Such networks consist of nodes (points) and links (arcs/edges). Nodes represent concepts and links represent connections between concepts. Concept mapping has been used for a variety of purposes, including to communicate complicated ideas and, as in the Telemakus system, to demonstrate connections among research findings.

## Methods

How might one apply the theories previously described in developing a comprehensive "real world" information retrieval and knowledge discovery system? As reviewed in the previous section, the Telemakus system is built on and extends prior research in the areas of concept representation, schema theory and information visualization. Work on components of what has become the Telemakus system has been underway for many years with a particular emphasis on the importance and utility of relationships extracted from data tables and figures [24,42-44]. Fuller [12,13] identified key objective elements important in representing a clinical research report and developed a schematic representation. The clinical trials schema has been adapted for representing basic sciences research reports in the Telemakus system.

Based on how scientists use and want to use the research literature, Telemakus brings together three innovations in analyzing, displaying and summarizing research reports across a domain:

1. Research Report Schema: Research methods and findings are extracted and presented in a consistent, coherent and structured schema format which mimics the research process itself and provides a high-level research report surrogate to facilitate searching as well as rapid review of retrieved documents.

2. Research Findings extracted from data tables and figures are used to index the documents, allowing searchers to request research studies which report a relationship between two concepts of interest.

3. Visual Exploration Interface provides a dynamic map of extracted research findings to graphically display what is known as well as, through gaps in the map, what is yet to be tested.

### Knowledgebase Creation & Components

The Telemakus system consists of a database, research report schema and tools to create relationship maps among concepts across documents. The research report schema serves as a surrogate for the study, methods and research findings for each document as well as providing an interactive search interface. The schematic representations include standard bibliographic information (author, title, journal), information about the research design and methods (age, sex, number of subjects, pre-treatment and treatment regimen, organism and source of organism) and, most importantly, research findings derived from data tables and figures.

The elements extracted by the Telemakus system from full-text documents are listed in Table 1. There are 22 fields for each document, with 12 routinely obtained from PubMed. Of the remaining fields, entries to 4 are controlled by thesauri. Two fields, Authors and SourceOfOrganisms use customized thesauri developed specifically for the Telemakus system. Two additional fields, the ResearchFindings and Organism fields, use the Unified Medical Language System^®^ (UMLS^®^) Metathesaurus^®^ as the basis for creating a controlled vocabulary.

**Table 1:** Research report schema database fields

**FieldName**	**Source**	**Comment**	**Thesaurus?**
RecordID	system provided	unique identifier	
Author	bibliographic database		Y
Year	bibliographic database		
Title	bibliographic database		
AuthorAddress	bibliographic database	author's email address	
Journal	bibliographic database		
Volume	bibliographic database		
Issue	bibliographic database		
Pages	bibliographic database		
Keywords	bibliographic database	subject headings	
Abstract	bibliographic database	entire abstract	
Tables Figures	document extracted	captions & URLs of tables/figures	
ResearchFindings	document extracted	pairs of related concepts	Y (UMLS)
Organism	document extracted	type of experimental subject	Y (UMLS)
Age	document extracted		
Sex	document extracted		
Pre-Treatment Characteristics	document extracted		
NumberOfSubjects	document extracted		
TreatmentRegimen	document extracted		
SourceOfOrganisms	document extracted		Y
AbstractURL	bibliographic database		
FullTextURL	bibliographic database	URL of online article	

The UMLS Metathesaurus is a rich database of information on concepts that appear in one or more of a number of different controlled vocabularies and classifications used in the field of biomedicine. It provides a uniform, integrated distribution format of over 95 biomedical vocabularies and classifications and contains syntactic information. All Metathesaurus concepts are assigned to specific types or categories – e.g., "Disease or Syndrome," "Virus" – and the Semantic Network contains information about the permissible relationships among these types – e.g., "Virus" causes "Disease or Syndrome" [45]. The 2004 edition of the UMLS Metathesaurus includes over 1 million biomedical concepts and 2.8 million concept names in its source vocabularies [46].

The thesauri are reviewed (curated by expert indexers) in order to create a consistent controlled vocabulary structure. As indicated in Table 1, research concepts and organism type thesauri are derived from the UMLS. As new concepts are identified from the document's data tables and figures, the UMLS is used to identify preferred terms that are added to the controlled vocabulary database. In addition to the preferred term, its synonyms, semantic type, broader and narrower terms and Unique Identifier are captured. The UMLS provides a very powerful approach to rapidly creating a robust scientific thesaurus in support of consistent and precise searching. Further, the semantic type descriptors for each concept and semantic network may offer some interesting opportunities for intelligent searching and mapping of research findings and their relationships in the future.

At present, data extraction utilizes systems with both manual and automated processes. An evolving thesauri-building and revising approach are important components of the Telemakus system to ensure that vocabulary identification and management reflect the specialized needs of the knowledge domain as new research concepts are identified and reported.

The knowledgebase construction process begins with an Internet search of a bibliographic database (e.g., PubMed, Web of Science^®^, etc.). Database elements are extracted and verified against the relevant thesaurus. As new concepts are identified the UMLS is checked for the preferred term and it is added to the appropriate Telemakus thesaurus – along with synonyms, narrower and broader terms.

**Figure 1:**
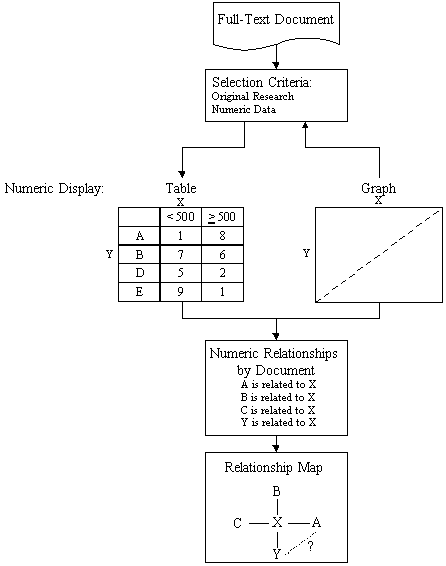
Process for deriving research relationships from data tables

One of the key innovations in the Telemakus system is the use of the data tables and figures for locating the concepts studied (and tested) by the researchers. Concentrating on the legends from data tables and figures focuses the extraction process and reduces the background noise of the full-text document, making the process tractable. In general, the information content of data tables and figures can be broken into two types: "facts" and "findings." Facts include reporting experimental design and comparative characteristics of animals in the study group (e.g., weight, age, pre-existing conditions, etc.). Findings are the results of the study (the research findings). Research findings are extracted from each of the "findings" data tables in a process described in Figure 1.

Table 2 provides a list of legends (the descriptions of content) from data tables and figures from a single research report and the end results of the extraction process. The legends are categorized into information content type (Fact or Research Finding), extracted concept relationships and concept relationships normalized (preferred terms) using the UMLS tools. In Table 2, the first two legends report "facts" (the experimental design and the composition of the diets of the research animals) while "findings" are reported in the remaining legends. The third column displays the noun phrases extracted from the legends which are then mapped to their corresponding UMLS preferred terms, as seen in the fourth column. When mapped, the term "dietary intake" maps to "energy intake" and "mammary gland carcinomas" maps to "mammary neoplasms." This provides a "controlled vocabulary" which enhances the consistency of retrieval from the knowledgebase.

**Table 2:** Information content type categorization and relationship concept candidates for a sample of table/figure legends Extracted from – Zhu Z, Haegele AD, Thompson HJ: Effect of caloric restriction on pre-malignant and malignant stages of mammary carcinogenesis. *Carcinogenesis* 1997, 18*(5)*:1007–12

**Table/Figure Legend**	**Type**	**Extracted Concept Relationships**	**Concept Relationships (after Normalization using UMLS tools)**
Table I. Sequence of events that comprised the experimental design	FACT	None	none
Table II. Composition of diets	FACT	None	none
Fig 1. Effect of caloric restriction on dietary intake, body weight gain and the ratio of cumulative body weight/cumulative diet intake.	FINDING	• dietary intake – caloric restriction • body weight – caloric restriction	• energy intake – caloric restriction • body weight – caloric restriction
Table III. Effect of calorie restriction on the proportion of intraductal proliferations, ductal carcinoma in situ and carcinomas in mammary glands	FINDING	• intraductal proliferations – caloric restriction • ductal carcinoma in situ – caloric restriction • mammary gland carcinomas – caloric restriction	• intraduct carcinoma of breast – caloric restriction • mammary neoplasms – caloric restriction
Fig 2. Effect of calorie restriction on cumulative and final incidences of mammary carcinomas.	FINDING	• mammary carcinomas – caloric restriction	• mammary neoplasms – caloric restriction
Fig 3. Percentage distribution of lesions in a dietary group that were: intraductal proliferations, ductal carcinoma in situ and adenocarcinoma.	FINDING	• lesions – dietary group • dietary group – intraductal proliferations • dietary group – ductal carcinoma in situ • dietary group – adenocarcinoma	• lesions – energy intake • intraduct carcinoma of breast – energy intake • adenocarcinoma – energy intake
Fig 4. Effect of caloric restriction on urinary excretion of immunoreactive cortical steroid.	FINDING	• immunoreactive cortical steroid – caloric restriction	• adrenal cortex hormones – caloric restriction

A current focus is the application of natural language processing (NLP) techniques to assist in the automation of concept extraction process. MetaMap, a program developed by the National Library of Medicine^®^, (NLM^®^) is being tested as a means of automatically parsing the legends from the data tables and figures to identify preferred UMLS concepts for addition to the Telemakus thesauri. MetaMap maps arbitrary text to concepts in the UMLS Metathesaurus; or, equivalently, it discovers Metathesaurus concepts in text. With this software, text is processed through a series of modules. First it is parsed into components including sentences, paragraphs, phrases, lexical elements and tokens. Variants are generated from the resulting phrases. Candidate concepts from the UMLS Metathesaurus are retrieved and evaluated against the phrases. The best of the candidates are organized into a final mapping in such a way as to best cover the text [47].

### Telemakus KnowledgeBase System Architecture

The Telemakus system architecture centers on: a relational database; a set of tools used to populate the knowledgebase with data extracted from bibliographic databases and full-text research reports; and several server side tools and programs responsible for delivering the content of the database to the public via the WWW. The entire system is built from open-source components, leveraging standard protocols and tools whenever possible.

The document processing system is initiated by an analyst who runs, reviews and edits as necessary extractions from the document being processed. It currently consists of a number of discrete phases to download, extract and analyze each document. These services are built primarily in Java running behind Tomcat and Apache and accessed by the analyst through the browser.

For the public Telemakus website interface, a number of open-source solutions have been selected and configured. An Apache web server intercepts all requests and delegates them to surrogate processes dedicated to each respective task. For requests to display the data from the database, the request is delegated to Zope, a content management service, for responding to the user's request. This typically includes running SQL queries against a PostgreSQL database and rendering the results in the conceptual schema that serves as a surrogate for each document. For tasks beyond simple queries and HTML requests, a Java Servlet™ is employed. As plain HTML is insufficient to effectively display and interact with the relationship map, a Java™ applet, TouchGraph, is used.

TouchGraph is an open-source concept-mapping tool for creating and navigating links between information sources. The tool was chosen for Telemakus because of its flexibility, customizing capabilities, high quality source code and compatibility with most browsers and operating systems (OS). The TouchGraph visualization package serializes maps to and from XML. By using Java, HTTP and XML, TouchGraph makes it easy to dynamically feed content to generate interactive nodes-and-edges maps.

### Database Query and Navigation: How Does it Work?

**Figure 2:**
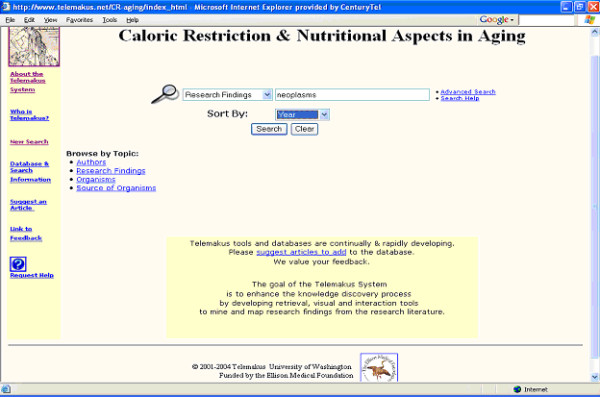
Telemakus search screen

Figure 2 shows the initial search screen – the starting point for a search of a knowledgebase. The user can search using Boolean logic on a number of fields, including the abstract, keywords, full-text, title, research findings, etc. Each of the thesauri – Author, Research Findings, Organisms and Source of Organisms – are also available for browsing and are directly searchable. Sorting is supported by year, first author, journal title.

**Figure 3:**
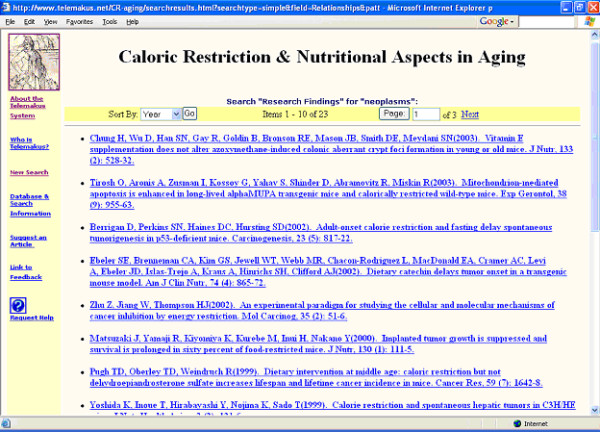
Display of retrieval set for a search on "neoplasms" (part 1)

**Figure 4:**
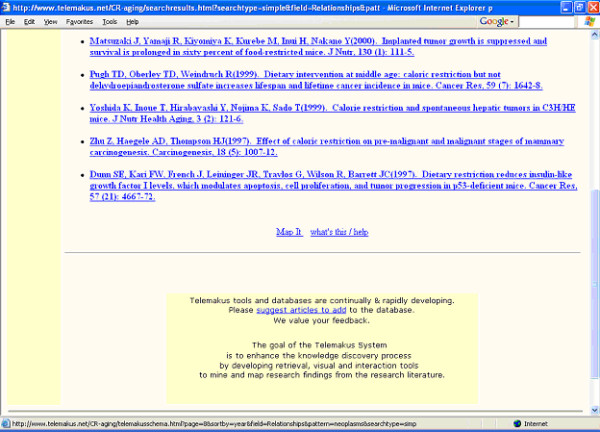
Display of retrieval set for a search on "neoplasms" (part 2)

**Figure 5:**
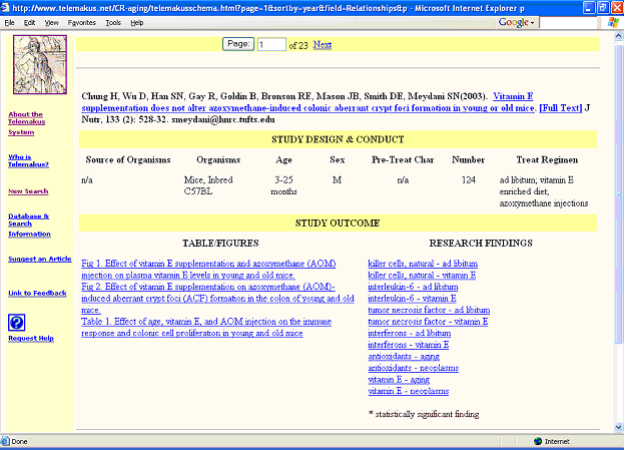
**Research report schema** Schema for one of the retrieved scientific reports from a search on caloric restriction and neoplasms

Figures 3 and 4 show the results of the search. Clicking on the first listing (Chung) results in the retrieval of the complete record for that item in the research report schema format (Figure 5), a rapid summary of research methods and organism characteristics that provides quick links to a variety of types of information including the full-text of the research article. Clicking on any blue highlighted item under "Table/Figure" takes the searcher to the respective figure in the full-text article.

The research report schema also serves as a convenient interface for searching for related research concepts, offering a rapid way of following research connections through the database. For instance, clicking on "killer cells, natural – ad libitum" would retrieve additional articles that present data tables linking those two concepts.

**Figure 6:**
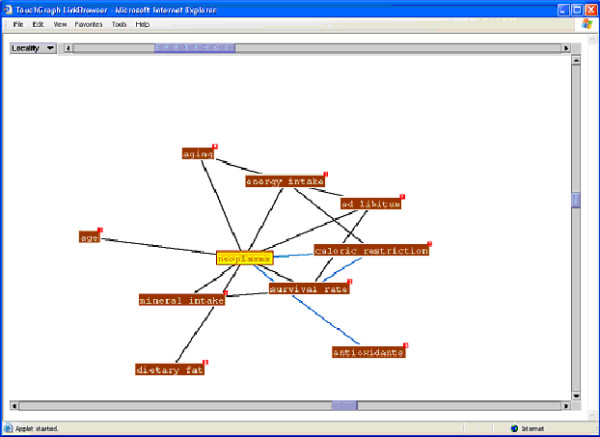
Concept map of research findings linked to neoplasms

**Figure 7:**
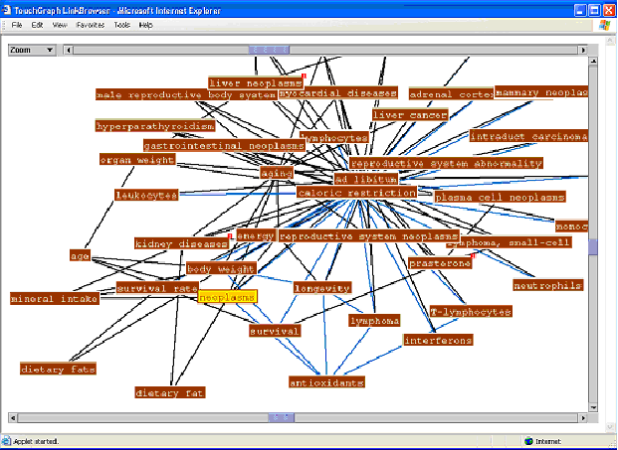
Expanded concept map of research findings relationships

The "map it" function, at the bottom of the retrieval set (Figure 4) provides access to the visualized maps of research findings connections for the current retrieval set. Examples of the concept maps generated by clicking on "map it" from Figure 4 are presented in Figures 6 and 7. Figure 6 presents a subset, more focused, map of research findings relating to the research concept of interest. Blue links highlight a reported (by the authors of the research report) statistically significant finding. The visualization tool permits moving from link to link and expanding the view to include a map of all research relationships reported in the retrieved set of documents (Figures 3 and 4). The user can also initiate a new search of a research term or link of interest (e.g., the relationship between survival rate and antioxidants) to retrieve all research papers which have reported this linkage. The iterative nature of the search process and ability to explore research connections from both the research schema as well as the research concept map supports the process of hypothesis exploration in a way that mimics the way many scientists work-by providing a means of exploring a variety of types of connections.

## Results

The first completed Telemakus knowledgebase focuses on caloric restriction in aging and is freely available at . Caloric restriction was an ideal starting point for Telemakus because it is an important and rapidly expanding specialized area of the biology of aging that is also highly interdisciplinary. Telemakus is a component of the Science of Aging (SAGE) project funded by the Ellison Medical Foundation. Other SAGE partners include the American Association for the Advancement of Science and Highwire Press, Stanford University (SAGE Knowledge Environment web site: ).

Formal usability testing of Telemakus is underway and will be the subject of a future article. Because a major goal of the Telemakus research program is to study scientists' approaches and preferences for accessing and using the scientific literature in order to create models and approaches for user-centered knowledgebases, researchers have been involved in the iterative design and testing of the system from its inception. The primary goals of this evaluation are to:

1. Determine scientists' preferences for working with the research literature.

2. Model preferred features based on those preferences.

3. Test the completeness of schema elements and structure as a document surrogate.

4. Experiment with and identify optimal visual representations to meet user needs.

5. Iteratively review/evaluate/test for improved performance in response to user feedback.

6. Identify domain(s) for future knowledgebase creation.

In general, response to each successive iteration of Telemakus has been positive and included constructive feedback for system enhancements and expansions. User feedback affirms that retrieval based on research findings is a unique and highly desirable core function. Further, the Telemakus schematic document surrogate has been enthusiastically received as a major improvement over the traditional citation format with abstract. As one researcher stated (and several others have echoed), "The strengths of Telemakus are doing what PubMed does not do, which is to give an outline of the main points and to allow searching off the figure/table legends, organisms/sources and outcome fields."

Additional feedback relates to the labeling of concept relationships as "statistically significant." Some researchers are interested in knowing the level of reported significance (i.e., p value) and asked for a detailed labeling to document this. In addition, there have been requests to consider labeling the relationships (i.e., directionality, type, etc.). Early testing of the mapping function resulted in the observation that color-blind individuals would not be able to see lines that were labeled with red or green, which led to a change in the mapping color scheme.

There have been many additional suggestions for improving the visualization, including addition of three-dimensional representations and allowing more user control of the presentation itself. Some researchers have expressed interest in being able to build maps based on the date a particular research finding was reported. This functionality would create time sequence maps that show the progression of research over time and, perhaps, will demonstrate paths of research that have been discarded prematurely and may be worth re-visiting. A number of researchers have indicated the utility of this approach for teaching purposes – for a student to quickly get a sense of the research "facts" in a domain. There have also been requests for tools to support downloading subsets of the knowledgebases, as well as tools to allow individuals to manipulate maps and add their own research findings and ideas to the concept maps.

## Discussion

While initial Telemakus development has focused on the research literature related to caloric restriction and the biology of aging, the goal is to expand into additional domains. For example, tables of genetic sequence information, which display reported relationships between gene sequences and diseases, are a natural area of expansion for Telemakus. There is great potential for building linkages between Telemakus knowledgebases and other factual databases, e.g., NCBI entrez resources. In addition, scientists from other domains beyond biomedicine (statistics, environmental research) have indicated that a customized schematic representation of research findings could be very useful in their domains.

Speeding up document processing so Telemakus can easily and efficiently scale for comprehensive treatment of domains is a key priority. As discussed previously, the UMLS Metathesaurus resources (in particular, MetaMap) are proving extremely useful. In addition, the Semantic Network will be tested for enhancing searching and visualization of research findings.

We will continue to utilize an iterative development method so that results of usability evaluation can immediately inform development of additional features. In particular, we want to test our hypothesis that the mapping feature will promote knowledge discovery by showing graphically what is known as well as, through lack of links, what research linkages have not yet been tested.

Since basic sciences researchers tend to initially focus on the data found within a report's tables and figures (sometimes before or instead of actually reading the article), extracting the headings and providing linked research concepts mimics a researcher's traditional approach to reading the research literature [42,48]. When users understand regularities in information spaces (layout, structure, landmarks, etc.) as schemata they acquire navigational knowledge in the form of a cognitive map of the information space. By providing the conceptual schema based on the scientist's own view of scientific research as a document surrogate, Telemakus provides a roadmap for reading and rapidly browsing through numerous research reports and aids in acquisition of the navigational knowledge required for a user to successfully explore complex information spaces [49].

One of the long-term goals of the Telemakus system is not to build knowledgebases "ad infinitum" but rather to create flexible tools for users to quickly and efficiently locate and visualize aggregate research findings from any domain which reports research findings as data. As more and more full-text research reports are available on the Internet, we believe the tools we are developing will provide an important approach for focusing on research findings and providing visual cues for quick review and assimilation.

## Conclusions

The Telemakus KnowledgeBase System builds on a good deal of prior research in a variety of domains. It provides a flexible new approach for creating knowledgebases to facilitate retrieval and review of scientific research reports. In formalizing the representation of the research methods and results of scientific reports, Telemakus offers a potential strategy to enhance the scientific discovery process. While other research has demonstrated that aggregating and analyzing research findings across domains augments knowledge discovery, the Telemakus system is unique in combining informative document representations with interactive concept maps of linked relationships across groups of research reports. Telemakus presents a novel approach to creating useful and precise document surrogates and may re-conceptualize the way we currently represent, retrieve and assimilate research findings from the published literature.

## Competing interests

None declared.

## Authors' contributions

SF conceived the study and contributed to its design, coordination and evaluation. DR and PB participated in the design of the study. DR led the overall coordination and drafted the manuscript. PB led the technical implementation. GMM contributed to the design, coordination and evaluation. All authors read and approved the final manuscript.
